# HIV viral load assessment in multiple sanctuaries – case presentation

**DOI:** 10.1186/1471-2334-14-S7-P35

**Published:** 2014-10-15

**Authors:** Anca Streinu-Cercel, Oana Săndulescu, Liliana Lucia Preoțescu, Dana Mihaela Jianu, Alina Cristina Neguț, Dragoş Florea, Dan Oțelea, Adrian Streinu-Cercel

**Affiliations:** 1 Carol Davila University of Medicine and Pharmacy, Bucharest, Romania; 2 National Institute for Infectious Diseases "Prof. Dr. Matei Balş", Bucharest, Romania; 3 Proestetica Medical Center, Bucharest, Romania

## Background

In our era, the profile of the HIV-infected patient has completely changed. We are now treating poly-experienced patients interested in their quality of life, where management of drug-associated adverse reactions and eradication of viral reservoirs constitute important issues.

## Case report

We present the case of a 57 year-old male patient, diagnosed with HIV infection for 14 years, and poly-experienced to antiretroviral (ARV) therapy (history of over 15 past ARV regimens). Early on in the evolution of HIV infection, the patient developed arterial hypertension, cardiomyopathy, 80% bi-carotid atheromatosis, and neurocognitive impairment.

The patient also developed dyslipidemia and significant lipodystrophy correlated with the use of first generation protease inhibitors. The impressive size of the buffalo hump led to impaired respiratory movements and trachea compression (identified through bronchoscopy, which had been indicated for the differential diagnosis of lung cavities; unfortunately because of the tracheal compression, bronchoscopy could not be performed). A surgical intervention for lipodystrophy had to be deferred because of the inability to maintain a stable ARV regimen, with undetectable viral loads. In Sep 2010 we managed to establish a stable ARV regimen, with boosted darunavir plus etravirine plus raltegravir. Plasma viral load decreased slowly, but it fluctuated, and it was only in Jan 2013 that the patient reached stable undetectable levels, and at this point, his CD4 cell count had increased to 321 cells/cmm.

In early 2014, after the patient had been virally suppressed for approximately one year, he was referred to a plastic surgery clinic, where he underwent successful lipoaspiration of 12 kg of adipose tissue from the buffalo hump, along with reconstruction of adipose facial features. Thus, the surgical procedure had a double role, both esthetic and functional, leading to improved pulmonary ventilation and a better tolerability to performing daily activities.

Given the history of difficulty maintaining a suppressed viral load in his patient, we assessed the potential HIV compartmentalization: after 6 months of consistently undetectable viremia, we performed sperm viral load (undetectable) and CSF viral load (undetectable), and after one year of viral suppression, we performed a viral load from the adipose tissue (results still in process).

## Conclusion

In patients with a long history of HIV infection and ARV therapy, there is need for close monitoring and interdisciplinary management of comorbidities, as well as viral load evaluation of HIV sanctuaries, where possible.

## Consent

Written informed consent was obtained from the patient for publication of this Case report and any accompanying images. A copy of the written consent is available for review by the Editor of this journal.

